# RETRACTION: Wound Infection and Healing in Minimally Invasive Plate Osteosynthesis Compared With Intramedullary Nail for Distal Tibial Fractures: A Meta‐Analysis

**DOI:** 10.1111/iwj.70264

**Published:** 2025-02-25

## Abstract

**RETRACTION:** WangS.‐F. and JiQ.‐L. , “Wound Infection and Healing in Minimally Invasive Plate Osteosynthesis Compared With Intramedullary Nail for Distal Tibial Fractures: A Meta‐Analysis,” International Wound Journal 21, no. 3 (2024): e14715, 10.1111/iwj.14715.38494179 PMC10944691

The above article, published online on 17 March 2024, in Wiley Online Library (http://onlinelibrary.wiley.com/), has been retracted by agreement between the journal Editor in Chief, Professor Keith Harding; and John Wiley & Sons Ltd. Following an investigation by the publisher, all parties have concluded that this article was accepted solely on the basis of a compromised peer review process. The editors have therefore decided to retract the article. The authors did not respond to our notice regarding the retraction.

**RETRACTION:** S.‐F. Wang and Q.‐L. Ji , “Wound Infection and Healing in Minimally Invasive Plate Osteosynthesis Compared With Intramedullary Nail for Distal Tibial Fractures: A Meta‐Analysis,” International Wound Journal 21, no. 3 (2024): e14715, 10.1111/iwj.14715.38494179 PMC10944691

The above article, published online on 17 March 2024, in Wiley Online Library (http://onlinelibrary.wiley.com/), has been retracted by agreement between the journal Editor in Chief, Professor Keith Harding; and John Wiley & Sons Ltd. Following an investigation by the publisher, all parties have concluded that this article was accepted solely on the basis of a compromised peer review process. The editors have therefore decided to retract the article. The authors did not respond to our notice regarding the retraction.

